# Improved production of *lactiplantibacillus plantarum* RO30 exopolysaccharide (REPS) by optimization of process parameters through statistical experimental designs

**DOI:** 10.1186/s12866-023-03117-z

**Published:** 2023-11-22

**Authors:** Eman Ahmed Elmansy, Ebtsam M. Elkady, Mohsen S. Asker, Nagwa A. Abdallah, Bigad E. Khalil, Shaimaa k. Amer

**Affiliations:** 1 Microbial Biotechnology Department, Institute of Biotechnology Research, National Research Centre, El-Tahreer Street, Dokki, Cairo, Egypt; 2 Microbiology Department, Faculty of Science, Ain Shams University, Cairo, Egypt; 3 Microbial Genetics Department, Institute Of Biotechnology Research, National Research Centre, El-Tahreer Street, Dokki, Cairo, Egypt

**Keywords:** Probiotic lactic acid bacteria, Exopolysaccharide, Optimization, Response surface methodology

## Abstract

**Background:**

In investigating of (exopolysaccharide) EPS from unconventional sources, lactic acid bacteria have a vital role due to their generally recognized as safe (GRAS) status. EPSs have diverse applications such as drug delivery, antimicrobial activity, surgical implants, and many more in many sectors. Despite being important, the main hindrance to the commercial application of these significant biopolymers is low productivity. Therefore, this study primarily focuses on optimizing physio-chemical conditions to maximize the previously produced EPS from probiotic *Lactiplantibacillus plantarum* RO30 (*L. plantarum* RO30) using one factor at a time (OFAT) and method Response Surface Methodology (RSM).

**Results:**

The EPS obtained from *L. plantarum* RO30 named REPS. The medium formulation for REPS production using the OFAT method revealed that sucrose (20 g/L, beef extract (25 g/L), and ammonium sulfate at 4 g/L concentration were the optimum carbon, organic and inorganic nitrogen sources, and REPS yield was increased up to 9.11 ± 0.51 g/L. RSM experiments revealed that, a greatly significant quadratic polynomial attained from the Central Composite Design (CCD) model was fruitful for specifying the most favorable cultural conditions that have significant consequences on REPS yield. The maximal amount of REPS (10.32 g/L) was formed by: sucrose (40 g/L), beef extract (25 g/L), pH (5.5), incubation temperature (30 °C), and incubation period (72 h). A high closeness was obtained between the predicted and experimental values and it displayed the efficiency of the RSM.

**Conclusion:**

This study was conducted to reinforce REPS production in the probiotic LAB *L. plantarum* RO30 by utilizing various experimental parameters. The maximum REPS yield of 10.32 g/L was attained under the circumstances optimized in the study.

## Background

 Exopolysaccharides (EPS) are polymers made up of long chains of sugar molecules that possess a high molecular weight and vary in terms of their structures and properties [1]. These polysaccharides are produced as secondary metabolites by various microorganisms such as archaea, bacteria, and fungi [2]. Microbial EPS can either be attached to the cell surface or released into the surrounding environment during microbial growth and metabolism. EPS are further classified based on their structures into two categories: homopolysaccharides (HoPS) and heteropolysaccharides (HePS) [3]. HoPS consist of a single type of sugar residue monomer, while HePS are composed of two or more sugar residue monomers, and in some cases, other organic molecules as well. The global interest in the production and extraction of EPS is driven by its low toxicity, enhanced bioactivity, and eco-friendly nature. In addition, EPS possesses multiple advantageous functional and physicochemical properties, including excellent water solubility, water retention capacity, emulsification capability, and ability to promote flocculation. Consequently, EPS finds applications in drug delivery, flocculation processes, heavy metal removal, and food emulsification [4]. Notably, EPS also exhibits a range of beneficial biological activities that promote human health, such as antioxidant, antibacterial, hypoglycemic, immunomodulatory, and cholesterol-lowering effects. As a result, EPS has garnered growing research attention [5].

Lactic acid bacteria (LAB) are a group of microorganisms informed for their ability to synthesize EPS, with their final byproducts being generally recognized as safe (GRAS). These LABs find applications in different industries, notably the food [6] and pharmaceutical sectors [7]. When utilized as a starter cultures or probiotics, LABs are reported to have a significant impact on the rheology and texture of fermented foods [8]. Among the LAB, *Lactobacillus* sp. have gained prominence due to their versatility in applications, such as drug delivery, antimicrobial activity, surgical implants, wound healing prospects, and many more across various districts.

Currently, about 30 species of LAB, including *Lactobacillus rhamnosus, Lactobacillus helveticus* and *Streptococcus thermophilus* [3], have been discovered as EPS producers. However, their productivity typically falls within the range of 25–800 mg/L, which is insufficient for large-scale industrial production [9, 10]. For instance, *Lactiplantibacillus plantarum* MM89, isolated from human breast milk, produces EPS with excellent immunomodulatory properties and acid phosphatase activity. It also promotes cytokine production. However, the yield of extracellular EPS-MM89 is only 590 mg/L, limiting its potential for large-scale production [11].

To facilitate commercial application of EPS, several strategies have been developed and applied to improve EPS production, including strain improvement [12], medium optimization [10, 13].

One of the crucial aspects of the biosynthesis of EPS from *Lactobacillus* sp. is medium composition as different nutrients preferred by different microorganisms, culture conditions such as pH, temperature, and incubation period. By optimizing the components of the medium and physical growth circumstances, the synthesis of EPS can be enhanced [14]. Consistently, optimal growth conditions are outlined operating a one factor at a time approach (OFAT). Nevertheless, this manner is both time-consuming and high-priced in terms of materials and human resources. In the worst outcome, the interactions between variables are frequently missed, leading to a deceiving conclusion [15]. In preference, computational intelligence ways such as Response Surface Methodology (RSM) can be employed to assess the optimal operative conditions for the highest EPS production. The purpose of the RSM is to ascribe the relationship among the response and the independent variables by mathematical models and to optimize this correlation [16]. The Central Composite Design (CCD) with a quadratic model is popularly utilized in processes of optimization. This design demonstrates how different factors affect the studied domain and the influence of these variables on the results and optimization procedure [17].

In our previous study, the biologically active heteropolysaccharide (REPS) was produced from the probiotic *L. plantarum* RO30 isolated from Romi cheese. It displayed well in vitro probiotic properties. REPS was extracted and characterized. The existence of COO−, OH and amide groups corresponding to typical EPSs was confirmed via FTIR. It was constituted of glucuronic acid, mannose, glucose, and arabinose in a molar ratio of 2.2:0.1:0.5:0.1, respectively. The average molecular weight was 4.96 × 104 g/mol. Also, the findings of in vitro antioxidant activities suggest that *L. plantarum* RO30 REPS possesses different antioxidant mechanisms. In addition to its antioxidant activity, the REPS also demonstrated good in vivo wound healing performance gave support for the hopeful use of REPS for applications as a therapeutic representative for burn wound healing [18].

So, the current work aimed to modify the cultural conditions for REPS production from probiotic *L. plantarum* RO30 using RSM to maximize the yield of REPS. The data obtained in this study may help to understand the specific conditions that influence REPS yield to be applicable.

## Materials and methods

### Culture medium

For the production of EPSs from LAB the modified MRS media was selected from the literature. The modified MRS medium used for EPS experiment had the subsequent composition (g/L): sucrose, 50; peptone, 10; beef extract, 10; yeast extract, 5; Tween 80, 1; tri ammonium citrate, 2; sodium acetate, 5; MgSO_4_.7H_2_O, 0.2; MnSO_4_.7H_2_O, 0.05; K_2_HPO_4_, 2, pH (5.8 ± 0.2) Sterilization was carried out by heating for 15 min at 121 °C.

### Microorganism

The probiotic *Lactiplantibacillus plantarum* RO30 strain we previously reported in [18] was employed. Briefly, *L. plantarum* RO30 was isolated from Romi cheese sample from local market in Egypt on specified DeMan, Rogosa and Sharpe MRS medium (pH 5.8) was incubated at 35 °C for 24 h for the isolation of *Lactobacillus* sp. The strain was identified based on 16 S rRNA sequencing and morphological characteristics. The strain was preserved at − 80 °C with an MRS medium containing 20% glycerol [18].

### Preparation of inoculum

The bacterial starter was prepared in 250 ml Erlenmeyer flasks retaining 100 ml sterile MRS medium (18 h at 35°C). The 24 h-old culture, at the logarithmic stage of growth, with an optical density (620 nm) of 1.0, was used as the inoculum in all experiments. These cultures were used as inoculum at 1% (v/v) for all the experiments.

### EPS extraction

To extract the EPS, a protocol by Suryawanshi et al. (2019) [14] with minor modifications was followed. Initially, the MRS medium with 5% sucrose was inoculated with 1% of the overnight bacterial culture at an optical density (O.D) of 1.25 at 620 nm. The mixture was then incubated at 35ºC for 72 h. Subsequently, bacterial cells were separated by centrifugation at 4ºC, 4000 rpm in a refrigerated centrifuge (SIGMA 3–18 KS), and for 20 min. the resulted cell free supernatant was subjected to trichloroacetic acid (TCA) to attain a final concentration of 6% (w/v). Then centrifugation was performed again (4000 rpm at 4ºC for 20 min) to get rid of the precipitated proteins. The clear supernatant was neutralized with NaOH and the EPS was precipitated by adding 3-volumes of cold absolute ethanol and kept overnight at 4ºC. The precipitate was recovered by centrifugation at 4000 rpm for 10 min at 4ºC. The precipitate was dissolved in ultrapure water and dialyzed for 2 days at 4ºC against the distilled water (changed twice each day), using a dialysis membrane having a cut-off of 3.5 kDa. After dialysis, the EPS was re-precipitated with 3-volumes of cold ethanol, and left overnight at 4ºC. The precipitate was separated by centrifugation at 4000 rpm for 10 min at 4ºC, washed twice with acetone, and finally with diethyl ether. The precipitate was dried in the oven at 35ºC to a constant weight was achieved.

### Exopolysaccharide yield quantification

The yield of crude REPS was determined using the phenol–sulfate acid method with glucose as a standard [19]. To perform the assay, 1 mL of sample was mixed with 1 mL of 5% phenol solution (w/v), followed by the addition of 5 mL of 98% sulfuric acid. The mixture was stirred and incubated at room temperature for 20 min, and the absorbance was measured at 490 nm. The yield of crude EPS was calculated based on the obtained absorbance value using a glucose standard curve.

### Procedure optimization and experimental design

#### Single-factor experiment method

The preliminary screening was carried to select the major components of the medium for maximizing REPS synthesis by strain *L. plantarum* RO30. All the experiments were performed in triplicate to minimize deviation. Results were expressed as mean ± S.E. One-way ANOVA was used for the single-factor experiment in the optimization study. Statistical analysis was of generated by IBM SPSS statistics 28 software. ANOVA data with *p* < 0.05 were considered as statistically significant.

#### Effects of different carbon origins on REPS production from *L. Plantarum* RO30

Various carbon compounds were added to the MRS broth for observing their effects on REPS Production from *L. plantarum* RO30. Different carbon origins (lactose, glucose, sucrose, and galactose) were added individually at a concentration of 2% (w/v). The yield of crude REPS was determined using the phenol–sulfate acid method with glucose as a standard [19].

#### Effects of different organic nitrogen sources on REPS production from *L. Plantarum* RO30

The effect of different organic nitrogen sources were studied by adding them individually in the production medium. The organic nitrogen origins include yeast extract, beef extract, and peptone (25 g/L), and the mixture of (yeast extract, beef extract, and peptone) in the ratio 1:2:2. The method of determining REPS production was the same as above.

#### Effects of different inorganic nitrogen sources on REPS Production from *L. Plantarum* RO30

The study also investigated the impact of various inorganic nitrogen compounds, including ammonium sulfate, ammonium nitrate, ammonium chloride, triammonium citrate, sodium nitrite, and potassium nitrate, at a concentration of 2 g/L. The method for evaluating REPS production remained consistent with the aforementioned approach.

#### Effects of different concentrations of *ammonium sulfate* on REPS Production from *L. Plantarum* RO30

Also, the most efficient inorganic nitrogen source **(**ammonium sulfate) was used at diverse concentrations (0.0, 1.0, 2.0, 3.0, and 4.0 g/L) in the production medium to find out the suitable concentration for REPS production [6, 20]. The method of determining EPS production was the same as above.

All experiments were established at 35ºC. The preliminary screening results would serve as a basis for the RSM experiment.

#### Center Composite Design (CCD)

 Relying on the results of initial OFAT experiments, the central composite design matrix of RSM was operated to optimize the five factors, namely sucrose concentration (A), beef extract concentration (B), pH (C), incubation temperature (D), and incubation period (E), at three coded levels (-1, 0, + 1) for realizing the maximization of REPS yield. The high status was (+ 1), medium (0), and the low status (-1). It is essential to enclose center points as well (in which all factors are at their central values). To establish the experimental design and analyze the results, the Design-Expert software, trial version 11.0 (Stat-Ease Inc., Minneapolis, USA) was operated. A whole of 45 trials, encompassing 42 factorial points and 3 central points, were executed (Table 1) [6].

**Table 1:** Design of different trials of the RSM for independent variables

Run	Sucrose (g/l) (A)	Beef ext. (g/l) (B)	pH (C)	Incubation temperature (D)	Incubation period (day) (E)
**1**	10 (-1)	15 (-1)	7 (+1)	35 (+1)	5 (+1)
**2**	10 (-1)	15 (-1)	7 (+1)	25 (-1)	5 (+1)
**3**	25 (0)	35 (+1)	5.5 (0)	30 (0)	3 (0)
**4**	40 (+1)	35 (+1)	7 (+1)	35 (+1)	5 (+1)
**5**	10 (-1)	15 (-1)	4 (-1)	25 (-1)	5 (+1)
**6**	10 (-1)	15 (-1)	7 (+1)	35 (+1)	1 (-1)
**7**	40 (+1)	15 (-1)	7 (+1)	25 (-1)	5 (+1)
**8**	40 (+1)	35 (+1)	7 (+1)	25 (-1)	5 (+1)
**9**	10 (-1)	35 (+1)	7 (+1)	35 (+1)	5 (+1)
**10**	40 (+1)	35 (+1)	4 (-1)	25 (-1)	5 (+1)
**11**	25 (0)	25 (0)	5.5 (0)	25 (-1)	3 (0)
**12**	40 (+1)	35 (+1)	4 (-1)	35 (+1)	1 (-1)
**13**	40 (+1)	15 (-1)	7 (+1)	35 (+1)	1 (-1)
**14**	10 (-1)	15 (-1)	4 (-1)	35 (+1)	1 (-1)
**15**	40 (+1)	35 (+1)	4 (-1)	25 (-1)	1 (-1)
**16**	10 (-1)	15 (-1)	4 (-1)	25 (-1)	1 (-1)
**17**	10 (-1)	15 (-1)	7 (+1)	25 (-1)	1 (-1)
**18**	40 (+1)	15 (-1)	4 (-1)	25 (-1)	1 (-1)
**19**	25 (0)	15 (-1)	5.5 (0)	30 (0)	3 (0)
**20**	40 (+1)	15 (-1)	4 (-1)	35 (+1)	1 (-1)
**21**	25 (0)	25 (0)	7 (+1)	30 (0)	3 (0)
**22**	40 (+1)	15 (-1)	4 (-1)	25 (-1)	5 (+1)
**23**	25 (0)	25 (0)	5.5 (0)	30 (0)	5 (+1)
**24**	25 (0)	25 (0)	4 (-1)	30 (0)	3 (0)
**25**	25 (0)	25 (0)	5.5 (0)	30 (0)	3 (0)
**26**	40 (+1)	25 (0)	5.5 (0)	30(0)	3 (0)
**27**	25 (0)	25 (0)	5.5 (0)	30 (0)	3 (0)
**28**	10 (-1)	15 (-1)	4 (-1)	35 (+1)	5 (+1)
**29**	10 (-1)	35 (+1)	7 (+1)	25 (-1)	1 (-1)
**30**	40 (+1)	15 (-1)	4 (-1)	35 (+1)	5 (+1)
**31**	10 (-1)	35 (+1)	4 (-1)	35 (+1)	5 (+1)
**32**	40 (+1)	15 (-1)	7 (+1)	25 (-1)	1 (-1)
**33**	25 (0)	25 (0)	5.5 (0)	30 (0)	3 (0)
**34**	10 (-1)	35 (+1)	7 (+1)	35 (+1)	1 (-1)
**35**	10 (-1)	25 (0)	5.5 (0)	30 (0)	3 (0)
**36**	10 (-1)	35 (+1)	4 (-1)	35 (+1)	1 (-1)
**37**	25 (0)	25 (0)	5.5 (0)	35 (+1)	3 (0)
**38**	10 (-1)	35 (+1)	7 (+1)	25 (-1)	5 (+1)
**39**	40 (+1)	35 (+1)	4 (-1)	35 (+1)	5 (+1)
**40**	10 (-1)	35 (+1)	4 (-1)	25 (-1)	1 (-1)
**41**	40 (+1)	35 (+1)	7 (+1)	25 (-1)	1 (-1)
**42**	10 (-1)	35 (+1)	4 (-1)	25 (-1)	5 (+1)
**43**	40 (+1)	35 (+1)	7 (+1)	35 (+1)	1 (-1)
**44**	25 (0)	25 (0)	5.5 (0)	30 (0)	1 (-1)
**45**	40 (+1)	15 (-1)	7 (+1)	35 (+1)	5 (+1)

Depending on the CCD experimental data, a second-order polynomial model was established, which correlated the relationship between REPS yield and the independent variables. The relationship could be expressed by the following equation:\

$${\mathrm Y}_{\mathrm{Activity}}=\;\mathrm\beta0\;+\;\mathrm\beta1\mathrm X1\;+\;\mathrm\beta2\mathrm X2\;+\;\mathrm\beta3\mathrm X3\;+\;\mathrm\beta4\mathrm X4\;+\;\mathrm\beta5\mathrm X5\;+\;\mathrm\beta12\mathrm X1\mathrm X12\;+\;\mathrm\beta13\mathrm X1\mathrm X13\;+\;\mathrm\beta14\mathrm X1\mathrm X14\;+\;\mathrm\beta15\mathrm X1\mathrm X15\;+\;\mathrm\beta23\mathrm X2\mathrm X23\;+\;\mathrm\beta24\mathrm X2\mathrm X24\;+\;\mathrm\beta25\mathrm X2\mathrm X25\;+\;\mathrm\beta34\mathrm X3\mathrm X34\;+\;\mathrm\beta35\mathrm X3\mathrm X35\;+\;\mathrm\beta45\mathrm X4\mathrm X45\;+\;\mathrm\beta11\mathrm X1^2\;+\;\mathrm\beta22\mathrm X2^2\;+\;\mathrm\beta33\mathrm X3^2\;+\;\mathrm\beta44\mathrm X4^2\;+\;\mathrm\beta55\mathrm X5^2$$

where, Y is the expected response, X1, X2, X3, X4 and X5 are coded input variables which influence the response variable Y, β0 is the intercept term, β1, β2, β3, β4, and β5 are the linear coefficient, β11, β22, β33, β44, and β55 are the quadratic coefficient and β12, β13, β14, β15, β23, β24, β25, β34, β35, and β45 are the interaction coefficient.

### Statistical analysis of the model

The statistical analysis of the model was accomplished to assess the analysis of variance (ANOVA). The model equation statistical significance was completed by Fisher’s test value, and the proportion of variance described by the model was designated by the estimation of multiple coefficients for each variable. The quadratic models were manifested as contour plots (3D), and the response surface curves were created by utilizing the Design-Expert software, trial version 11.0 (Stat-Ease Inc., Minneapolis, USA). The degree of excellence of the polynomial model equation was forecasted employing the coefficient of determination (R^2^) and adjusted R^2^.The predicted optimal REPS yield and the experimentally optimal REPS yield were subsequently analyzed using T-test.

## Results

### Selection of the influential media components for process modeling the REPS production and data analysis

#### Carbon source

**Fig. 1:**
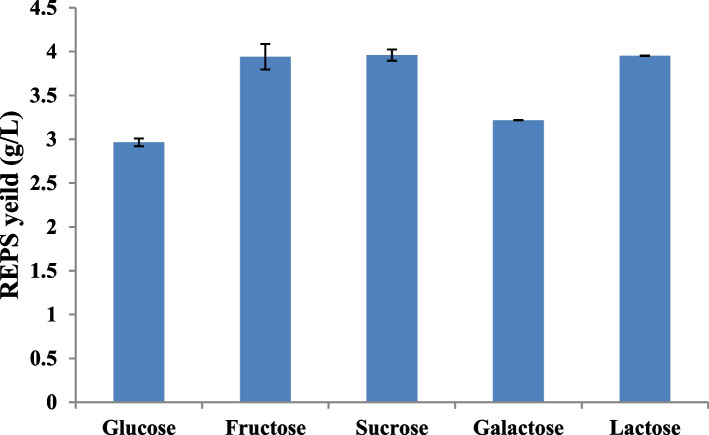
Effect of different carbon sources on REPS production by *L. plantarum *RO30. Results are presented as means ± SE for triplicate. Bars indicate standard errors

According to the ANOVA results, the interaction effects between different carbon sources and *L. plantarum* RO30 on the REPS concentration were found to be statistically significant (*P* < 0.001) Fig. (1). The supplemented sugars can be arranged as sucrose, lactose, fructose, galactose, and glucose in decreasing order of the EPS yield. Sucrose was more advantageous for REPS generation by *L. plantarum* RO30 (3.96 ± 0.0651 g/L). The lowest amount of REPS production was registered in glucose containing medium (2.97 ± 0.0441 g/L).

#### Organic nitrogen sources

**Fig. 2:**
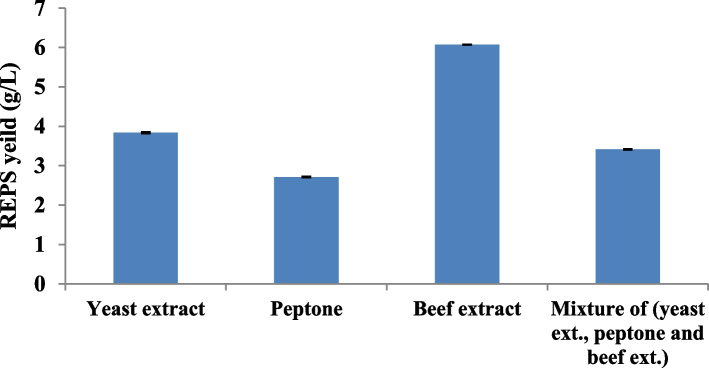
Effect of different organic nitrogen sources on REPS production by *L. plantarum *RO30. Results are presented as means ± SE for triplicate. Bars indicate standard errors

The various organic nitrogen sources used had a statistically significant (*p* < 0.001) impact on REPS production by *L. plantarum* RO30 as shown in Fig. (2). Among the tested organic nitrogen sources, the highest amount of REPS production was registered in beef extract containing medium (6.07 ± 0.008 g/L) as the sole nitrogen source. The lowest amount of REPS production was registered in peptone containing medium (2.71 ± 0.018 g/L).

#### Inorganic nitrogen source

**Fig. 3:**
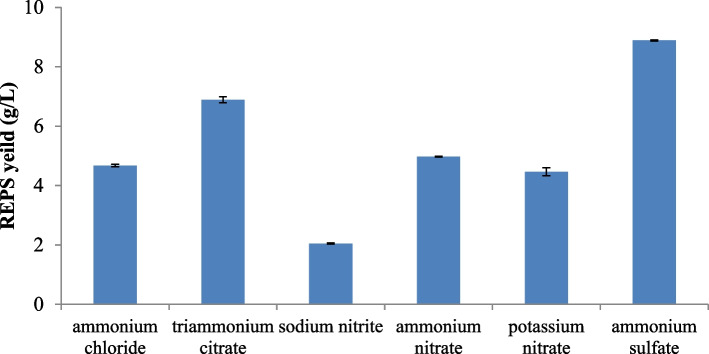
Effect of different inorganic sources of nitrogen on REPS production by *L. plantarum *RO30

The various inorganic nitrogen sources possessed a statistically significant impact on REPS production by *L. plantarum* RO30 as shown in Fig. (3) (*P* < 0.001). Among the tested organic nitrogen sources, the highest amount of REPS production was registered in ammonium sulfate containing medium (8.89 ± 0.018 g/L) as the sole inorganic nitrogen source. The lowest amount of REPS production was registered in a sodium nitrate containing medium (2.05 ± 0.017 g/L). Comparing the nitrogen supplements investigated in this experiment in terms of their effectiveness on REPS synthesis, they could be organized in descending sequence as ammonium sulfate, triammonium citrate, ammonium nitrate, ammonium chloride, potassium nitrate, and sodium nitrate.

#### Effect of different concentrations of (NH_4_)_2_SO_4_ on REPS biosynthesis by *L. plantarum *RO30

**Fig. 4:**
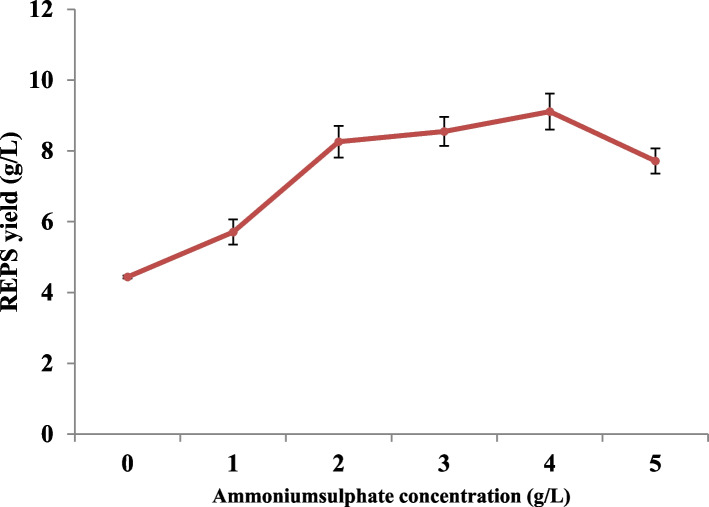
Effect of different concentrations of (NH_4_)_2_SO_4_ on REPS biosynthesis by *L. plantarum *RO30. Results are presented as means ± SE for triplicate. Bars indicate standard errors

Regarding the effect of different concentrations of (NH_4_)_2_SO_4_ on REPS yield by *L. plantarum *RO30, data in Figure (4) illustrated that when the concentration of (NH_4_)_2_SO_4_was enlarged the creation raised first and then diminished. The highest amount of REPS production was registered at a concentration of 4.0 g/L ammonium sulfate (9.11±0.51 g/L).

#### Optimization using RSM

 Sucrose, beef extract, and ammonium sulfate at 4.0 g/L concentration were chosen as the most prominent factors influencing REPS yield from OFAT experiments. The most appropriate incubation period, incubation temperature, pH, and optimum levels of the sucrose and beef extract and the effect of their interactions on REPS production were determined by CCD of RSM. Table 2 showed total forty-five runs based on the model generated by the software, the details of the actual and coded values employed in the RSM as well as the anticipated and observed responses for REPS production (Y). The difference in the REPS yield was observed during the 45 runs of the experiment. The alternation within the REPS production was owing to the different conditions in the experiment in each run, reflecting the importance of statistical optimization of fermentation condition over the traditional fermentation conditions. The results showed that the minimum and maximum REPS productivity were 5.3 and 10.32 g/L, respectively. The maximum REPS yield (10.32 g/L) was obtained after an optimized culture condition at sucrose 40 g/L, beef extract 25 g/L, pH 5.5, fermentation temperature 30 °C, and period of fermentation 72 h (Run 26).

**Table 2:** Central composite design matrix for RSM revealed observed and predicted output of REPS

Run	A: Sucrose (g/l)	B: Beef ext. (g/l)	C: pH	D: Incubation Temp. (ºc)	E: Incubation period (day)	REPS yield (g/l)		
						Actual	Predicted	Residual
**1**	10	15	7	35	5	5.51	5.55	-0.0404
**2**	10	15	7	25	5	5.36	5.4	-0.0374
**3**	25	35	5.5	30	3	7.86	7.89	-0.0269
**4**	40	35	7	35	5	7.74	7.77	-0.0334
**5**	10	15	4	25	5	5.47	5.46	0.0132
**6**	10	15	7	35	1	7.04	7.04	0.0021
**7**	40	15	7	25	5	6.81	6.84	-0.034
**8**	40	35	7	25	5	8.01	7.87	0.1421
**9**	10	35	7	35	5	6.59	6.63	-0.0368
**10**	40	35	4	25	5	7.49	7.53	-0.0398
**11**	25	25	5.5	25	3	7.5	7.52	-0.0222
**12**	40	35	4	35	1	8.53	8.53	-0.0002
**13**	40	15	7	35	1	8.12	8.12	-0.0044
**14**	10	15	4	35	1	7.1	7.06	0.0428
**15**	40	35	4	25	1	8.98	8.78	0.2027
**16**	10	15	4	25	1	7.03	7.06	-0.0268
**17**	10	15	7	25	1	7.09	7.05	0.0376
**18**	40	15	4	25	1	8.1	8.1	-0.0008
**19**	25	15	5.5	30	3	6.94	7.01	-0.0705
**20**	40	15	4	35	1	7.89	7.96	-0.0713
**21**	25	25	7	30	3	7.67	7.68	-0.0134
**22**	40	15	4	25	5	6.62	6.72	-0.1008
**23**	25	25	5.5	30	5	6.82	6.64	0.1811
**24**	25	25	4	30	3	7.44	7.52	-0.084
**25**	25	25	5.5	30	3	7.91	8.01	-0.0968
**26**	**40**	**25**	**5.5**	**30**	**3**	**10.32**	**10.01**	0.3137
**27**	25	25	5.5	30	3	8.3	8.01	0.2932
**28**	10	15	4	35	5	5.85	5.62	0.2253
**29**	10	35	7	25	1	8.32	8.1	0.2161
**30**	40	15	4	35	5	6.72	6.75	-0.0287
**31**	10	35	4	35	5	6.5	6.49	0.0138
**32**	40	15	7	25	1	8.45	8.28	0.171
**33**	25	25	5.5	30	3	8.2	8.01	0.1932
**34**	10	35	7	35	1	8.02	7.98	0.0381
**35**	10	25	5.5	30	3	8.5	8.91	-0.411
**36**	10	35	4	35	1	7.76	7.79	-0.0262
**37**	25	25	5.5	35	3	7.4	7.48	-0.0752
**38**	10	35	7	25	5	6.59	6.58	0.0086
**39**	40	35	4	35	5	7.35	7.45	-0.1002
**40**	10	35	4	25	1	7.93	7.89	0.0367
**41**	40	35	7	25	1	8.66	9.17	-0.5105
**42**	10	35	4	25	5	6.37	6.43	-0.0557
**43**	40	35	7	35	1	9.08	8.91	0.1716
**44**	25	25	5.5	30	1	7.73	8.01	-0.2785
**45**	40	15	7	35	5	6.78	6.86	-0.0769

The obtained actual results were modeled with the subsequent second-order polynomial equation to clarify the relationship among the yield of REPS and the observed variables:$$\begin{aligned} &\mathbf{REPS, Y (g/L)} = + 8.01 + 0.5476\ \text{A} + 0.4382\ \text{B} - 0.0797\ \text{C} - 0.0235\ \text{D} - 0.6838\ \text{E} - 0.04\ \text{AB} +\\ &0.0456\ \text{AC}\, -\, 0.035\ \text{AD}\, +\, 0.055\ \text{AE}\, +\, 0.0538\ \text{BC} - 0.0269\ \text{BD} + 0.0331\ \text{BE} - 0.0037\ \text{CD} -\\ &0.0137\ \text{CE} + 0.0419\ \text{DE} + 1.45\ \text{A}^{2} - 0.5582\ \text{B}^{2} - 0.4032\ \text{C}^{2} - 0.5082\ \text{D}^{2} - 0.6842\ \text{E}^{2}. \end{aligned}$$  

 The results of experimental data were subjected to analysis of variance (ANOVA), and statistical tests were performed with the F test shown in Table 3. ANOVA results for the RSM quadratic equation for the yield of REPS (Y, g/L) stipulated the ‘F-value’ to be 48.80, *p* < 0.0001, indicating that the regression model was statistically significant. There is only a 0.01% chance that F-value this large could happen because of noise. Model terms having values of ‘Prob > F’ less than 0.05 designated model terms are regarded significant, whereas those bigger than 0.10 are insignificant. In compliance with the current model A, B, C, E, A², B², C², D², and E² were significant model terms. That is, the linear effect of sucrose, beef extract, pH, incubation time, and the quadratic effect of sucrose, beef extract, pH, and temperature were significant. The lack of fit F-value of 1.08 insinuates the lack of fit is not significant in proportion to the pure error and that the model fits.

**Table 3:** Analysis of variance (ANOVA) results for the central composite design (CCD) quadratic model for the response

Source	Sum of Squares	d.f	Mean Square	F-value	***p***-value	
**Model**	43.05	20	2.15	48.8	< 0.0001	significant
A-sucrose	10.2	1	10.2	231.18	< 0.0001	
B-beef	6.53	1	6.53	148.04	< 0.0001	
C-pH	0.216	1	0.216	4.9	0.0367	
D-Temperature	0.0188	1	0.0188	0.4267	0.5198	
E-time	15.9	1	15.9	360.38	< 0.0001	
AB	0.0512	1	0.0512	1.16	0.292	
AC	0.0666	1	0.0666	1.51	0.231	
AD	0.0392	1	0.0392	0.8887	0.3552	
AE	0.0968	1	0.0968	2.19	0.1515	
BC	0.0925	1	0.0925	2.1	0.1606	
BD	0.0231	1	0.0231	0.524	0.4761	
BE	0.0351	1	0.0351	0.796	0.3811	
CD	0.0005	1	0.0005	0.0102	0.9204	
CE	0.0061	1	0.0061	0.1372	0.7144	
DE	0.0561	1	0.0561	1.27	0.2705	
A²	5.19	1	5.19	117.71	< 0.0001	
B²	0.7674	1	0.7674	17.4	0.0003	
C²	0.4004	1	0.4004	9.08	0.006	
D²	0.6361	1	0.6361	14.42	0.0009	
E²	1.15	1	1.15	26.14	< 0.0001	
**Residual**	1.06	24	0.0441			
Lack of Fit	0.9766	22	0.0444	1.08	0.5884	not significant
Pure Error	0.0821	2	0.041			
**Cor Total**	44.11	44				
**SD**	0.21		**R²**		0.976	
**Mean**	7.48		**Adjusted R²**		0.956	
**C.V. %**	2.81		**Predicted R²**		0.9143	
			**Adeq Precision**		32.1241	

The goodness of fit of the model was inspected by the determination coefficient (R^2^). In this case, ANOVA indicated the R^2^ value of 0.976 which is near to 1.0, which recommended that the second-order polynomial regression equation has a goodness of fit and reasonable concurrence in the response represented by the model. This once more confirmed a satisfactory adaptation of the quadratic model to the experimental data and indicated that this model could interpret 97% response variability. The adequate precision estimates the signal-to-noise ratio. A ratio more than 4 is greatly desired. The ratio of 32.1241 indicates an adequate signal. The ‘Pred R^2^’ of 0.9143 is in acceptable harmony with the ‘Adjusted R^2^’ of 0.9560 for Y. A good correlation between observed and predicted results reflected the precision and relevancy of the central composite design for the process of optimization.

The coefficient of variation (CV) indicates the degree of precision with which the experiments are compared. The decreased reliability of the experiment is usually indicated by the high value of CV. In the current situation value of CV (2.81) is lower than 10%, indicating that the model is good and can precisely predict the synthesis of REPS under the experimental factors, which shows that the mathematical model was applicable to the simulation of REPS biosynthesis in this study.

The probability value < 0.0001, < 0.0001, 0.0367, and < 0.0001, respectively for sucrose, beef extract, pH, and incubation period ensures the factors are significant in the REPS production.

**Fig. 5:**
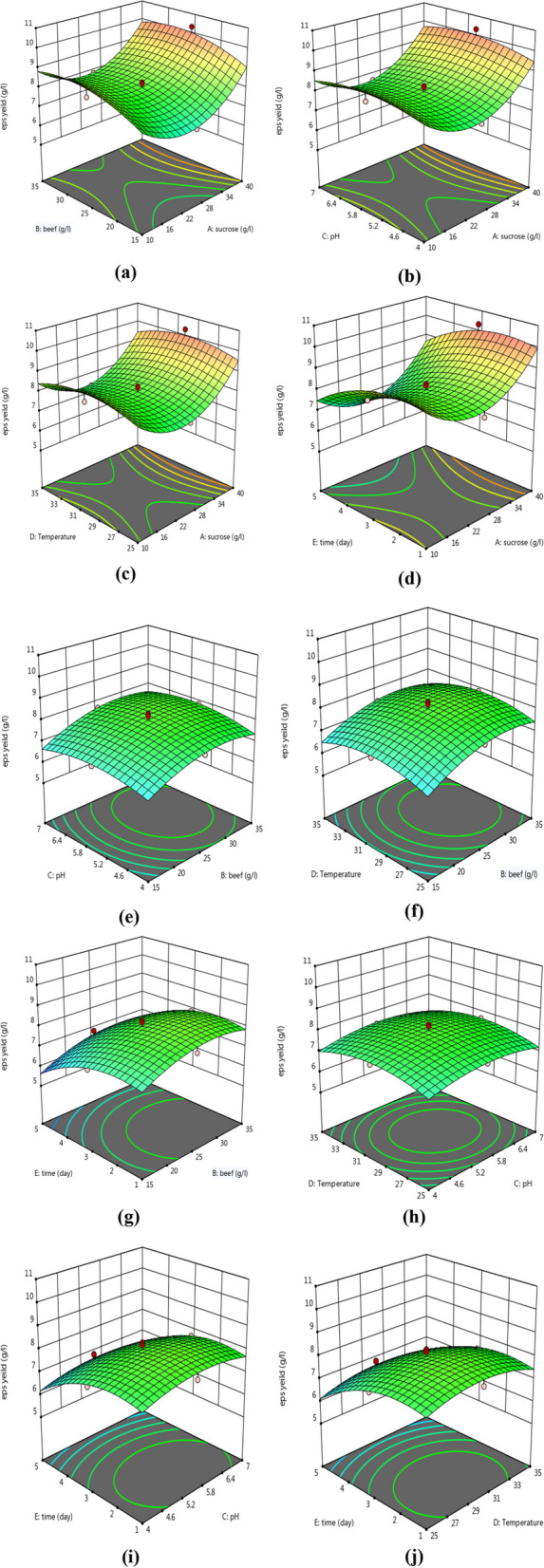
Response surface plot of the interaction effect of (**a**) sucrose, beef extract (**b**) sucrose, pH (**c**) sucrose, incubation temperature (**d**) sucrose, incubation period (**e**) beef extract, pH, (**f**) beef extract, incubation temperature, (**g**) beef extract, incubation period, (**h**) pH, incubation temperature, (**i**) pH, incubation period, and (**j**) incubation temperature, incubation period on REPS production by *L. plantarum* RO30

 However, the interactions between the factors were not found to be significant showing that the interaction between factors is not necessary for REPS production. Moreover, we could observe the interaction effects through the 3D response surface plots and contour plots of the factors in Fig. 5a.

**Fig. 6:**
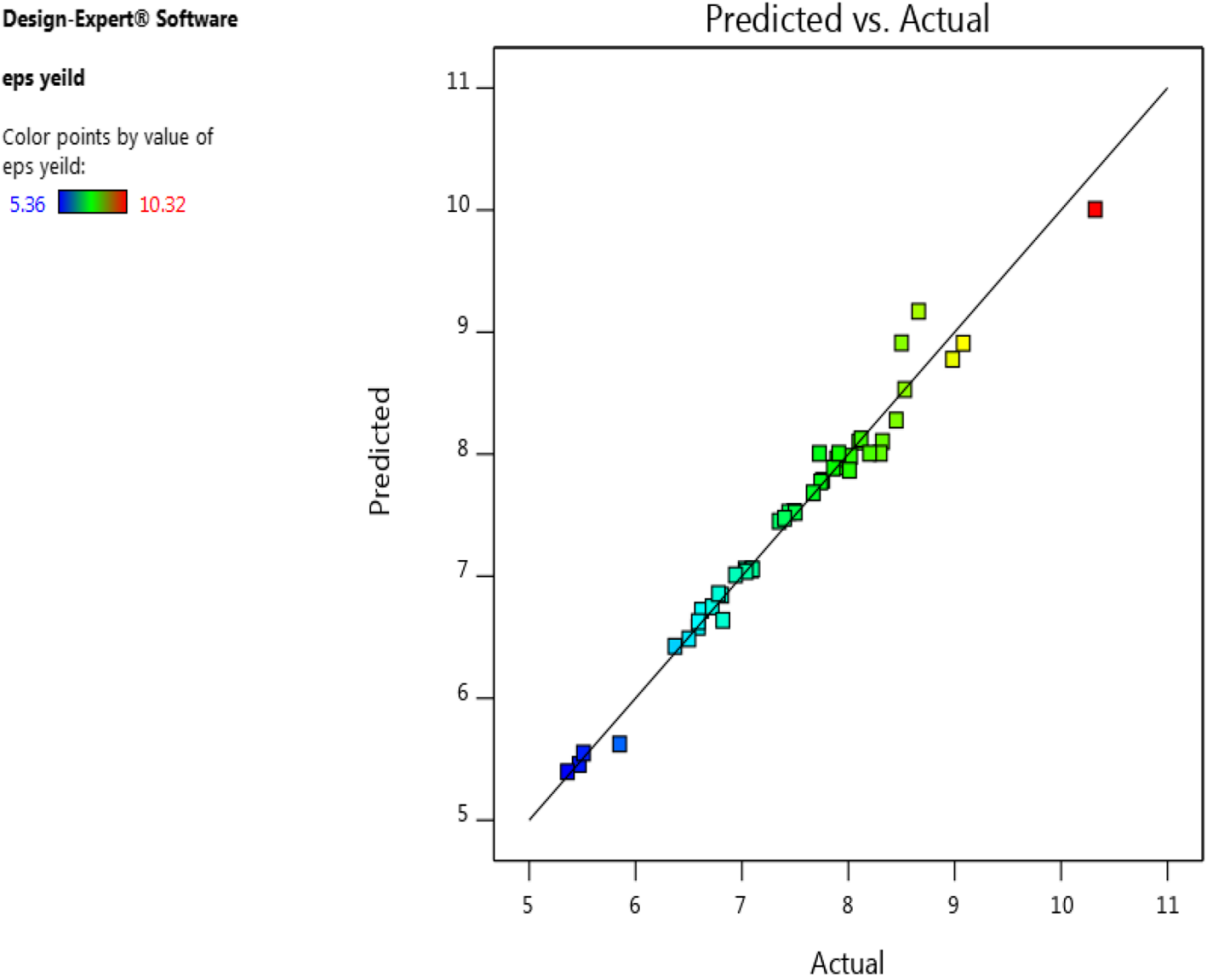
Predicted Vs. actual values of REPS production (g/L)

If predicted and obtained values lie on a straight line, it would refer to a good accordance of the predicted values and advocate the perfection of the model. The diversion of actual values from the observed values was plotted in Fig. (6). It was noticed that there were not many aberration in the experimental and predicted values revealing the significance of the model. The results of T-test (One-Sided *p* = 0.492, two-Sided *p* = 0.985) showed that there were no significant differenes between the predicted and actual REPS yield. Hence, the model was successfully validated.

## Discussion

Although the genetic background of the bacteria affects the composition and yield of EPS production, the total quantity of the EPSs are also strongly dependent upon the nutritional and physical culture circumstances [21]. Many researchers have investigated the total yield of EPS in different media and production surroundings [22, 23]. The single-factor experiment and response surface methodology were employed together to refine the medium components and culture conditions for REPS production.

The single-factor experiment initially identified the optimal conditions for REPS production were sucrose, beef extract, ammonium sulfate at 4 g/L concentration. Then the optimum concentrations of sucrose and beef extract, pH, incubation temperature, and the optimum incubation period, and their interactive effects on REPS production were further optimized by RSM. The optimal values of the tested variables were sucrose (40 g/L), beef extract (25 g/L), pH (5.5), growth temperature (30ºC), and period of incubation (72 h). Under these optimized conditions, the RSM model predicted a maximum REPS output of 10.01 g/L. In experimental (actual), the achieved REPS production amounted to 10.32 g/L.

Due to the variability in carbon metabolism pathways among lactic acid bacteria, it’s crucial to carefully select an appropriate carbon source to achieve optimum EPS production [24, 25]. Also, carbon sources greatly affect the chemical composition of LAB EPS [26]. In this study, the five carbon sources demonstrated a statistically significant effect on the yield of REPS, with sucrose emerging as the most effective carbon source for REPS production. However; studies have shown that different strains may have individual preferences for specific sources of carbon which can enhance efficient growth and EPS synthesis. For instance, sucrose was found to be more favorable than glucose for *L. plantarum* 70,810 EPS synthesis [25]. Also, Adesulu-Dahunsi et al. (2018) [26] found that sucrose (20 g/L) was the best sources of carbon for EPS production by *W. cibaria* GA44 (3.6 g/L). On the other hand, several studies have shown that glucose was an effective carbon source for EPS production by several LAB strains. Imran et al. (2016) [6] indicated that *L. plantarum* NTMI05 and NTMI20 strains exhibited higher EPS production in the presence of glucose, as compared with the other carbon sources tested (galactose, lactose, and sucrose). Also, Gancel and Novel (1994) [27] reported that the maximal synthesis of EPS in *Streptococcus salivarius* ssp. *thermophilus* S22 was relying upon lactose. This preference is enhanced by their biochemical or metabolic potential as well as their genetic makeup. LABs utilize various housekeeping enzymes such as glycosyl transferases, translocases, and polymerases to synthesize polysaccharides. These enzymes utilize sugars and other components in the medium to produce the polysaccharides [6].

The carbon source is mainly utilized for the production of energy required for cell growth and EPS production, as well as for the biosynthesis of biomass and EPS precursors. Although amino acids do not directly play role in EPS production, they may provide key carbon and nitrogen sources for the synthesis of essential cell components. Also, EPS is primarily conjugated with proteins, and nitrogen sources affect EPS synthesis by influencing the synthesis of these proteins [28, 29]. Moreover, the activity of enzymes involved in EPS biosynthesis can also be affected by nitrogenous sources [30]. So, they were found to significantly influence EPS synthesis. In this study, the beef extract positively increased the production of REPS more than others and this might be due to the fact that beef extract possesses numerous nutritional properties, and is constituted of a combination of peptides, amino acids, nucleotides, organic acids, minerals, phosphates, energy sources, and some vitamins. The incorporation of beef extract in the medium effectively promotes the growth and metabolism resulting in better enhanced functionality of the process [31, 32].

Besides carbon and nitrogen sources, inorganic salt also play a role in influencing the biosynthesis of LAB-derived EPS. Similar to our results, Ismail and Nampoothiri [33] demonstrated that ammonium-sulfate raised the EPS yield of *L. plantarum* MTCC 9510 strain positively, which was elevated from 140 to 1080 mg/L.

Medium pH may affect the growth rate and metabolic activity of LAB, and hence EPS production. Various metabolic or enzymatic activities required for EPS production may need an optimum pH; therefore, medium pH is an important process parameter [32]. Although the optimum pH for EPS formation has been found to vary depending on the LAB strain and the experimental conditions, it is generally around pH 6.0 [34, 35] which is almost similar with our results. Khanh and Thao (2016) [36] reported that *L. plantarum* T10 strain produced the highest EPS amount (397.72 mg/L) at pH 5.5, which was is in accordance with our results. Haj-Mustafa et al. (2015) [37] who worked on *L. rhamnosus* 519 reported that the maximum EPS amount was obtained at pH 5.7. Contrary to our results For *L. delbrueckii* subsp. *bulgaricus* B3 and G12 strains, the greatest amount of EPS was obtained at pH 6.2, as compared with the other pH values tested (pH 4.0, 4.5,5.0, 5.5, 6.0, and 7.0) [36]. Also, Imran et al. (2016) [6] indicated that the neutral pH promoted the EPS production in *L. plantarum* NTMI05 and NTMI20 strains.

The optimum fermentation temperature of 30ºC was interesting because of deviation from the optimum growth temperature of 35ºC for *L. plantarum* [18]. This was not the same as the majority of the relevant studies, which recorded the same temperature [1, 38, and 39One plausible hypothesis posits that microorganisms may increase EPS production as a protective response under adverse conditions [1, 40, and 41]. Supporting this hypothesis, the research conducted by Wang et al. [1], Bengoa et al. [13], and Oleksy-Sobczak et al. [42] revealed optimum temperatures of 27 ºC, 20ºC, and 25ºC, and respectively. Generally, mesophilic LAB (lactic acid bacteria) were reported to exhibit maximum EPS production around 25ºC [43]. The stimulatory effect of lower temperature on EPS production by mesophilic LAB can be attributed by the fact that under sub-optimal growth temperatures slower growing cells exhibit a slower biosynthesis of cell wall polymers. As a result, a greater proportion of isoprenoid lipid carrier precursors, such as undecaprenol (C55) lipid carrier, are used in the biosynthesis of EPS rather than for cell wall material production. This increased allocation of undecaprenol contributes to enhanced EPS production. Undecaprenol play a critical role in the biosynthesis of EPS as well as in the formation of cell wall components like peptidoglycan, lipopolysaccharide, and teichoic acid [13, 35, and 43].

The optimal incubation period is another important factor that may play an important role in determining the yield of EPS from a particular organism. In sufficient duration can lead to low yield of EPS, whereas excessively prolonged incubation can lead to degradation of EPS due to glycohydrolase activity, other reactions, resulting in a reduction in overall productivity [44]. In our study, the highest EPS production was attained after 72 h of incubation period, aligning with similar findings by Imran et al. (2016) [6] for *L. plantarum* NTMI05 and NTMI20.

The 3D response surface plots and contour plots of the factors showed that the interaction among the studied factors was not significantly different. Wang et al. (2023) [1] also reported that the interaction among the three factors (MgSO4 concentration was 0.01%, initial pH was 7.4, and inoculation size was 6.4%) was not significantly different (PAB = 0.2197, PAC = 0.9243, and PBC = 0.1457). However, the results attained in the current study revealed that the mathematical model was proficient for the reproduction of EPS. Above all, the optimum conditions for producing REPS increased it by about 2.5 folds. In the same way, Wang et al. (2017) [38] informed that EPS output from *L. plantarum* KX041 was successfully increased about 3-times than the initial production (0.599 g/L) utilizing RSM at maximum circumstances of soybean peptone (20 g/L), fermentation temperature (35 °C) and pH (6.38).

## Conclusion

In this study, the medium composition of modified MRS media was optimized with the conventional OFAT method and RSM approaches to enhance the production of REPS. REPS yield increased by about 2.5 folds and reached up to 10.32 (g/L). Similar optimization and modeling process may be employed in the future for the higher production of EPS for different applications from suitable microorganisms.

## Data Availability

All data generated or analysed during this study are included in this published article.

## Abbreviations

EPS: Exopolysaccharide
REPS: Exopolysaccharide extracted from *Lactiplantibacillus plantarum *RO30
LAB: Lactic Acid Bacteria
RSM: Response Surface Methodology
OFAT: One Factor at Time
CCD: Central Composite Design

## References

[1] Wang J, Zhang J, Guo H, Cheng Q, Abbas Z, Tong Y, Yang T, Zhou Y, Zhang H, Wei X, Si D, Zhang R (2023). Optimization of exopolysaccharide produced by *Lactobacillus plantarum* R301 and its antioxidant and anti-inflammatory activities. Foods.

[2] Zhao XZ, Liang QL (2023). Optimization, probiotic characteristics, and rheological properties of exopolysaccharides from lactiplantibacillus plantarum MC5. Molecules.

[3] Angelin J, Kavitha M (2020). Exopolysaccharides from probiotic bacteria and their health potential. Int J Biol Macromol.

[4] Devi PB, Kavitake D, Shetty PH (2016). Physico-chemical characterization of galactan exopolysaccharide produced by *Weissella confusa* KR780676. Int J Biol Macromol.

[5] Al-Nabulsi AA, Jaradat ZW, Al Qudsi FR, Elsalem L, Osaili TM, Olaimat AN, Esposito G, Liu SQ, Ayyash MM (2022). Characterization and bioactive properties of exopolysaccharides produced by Streptococcus thermophilus and Lactobacillus bulgaricus isolated from labaneh. LWT.

[6] Imran MYM, Reehana N, Jayaraj KA, Ahamed AAP, Dhanasekaran D, Thajuddin N, Alharbi NS, Muralitharan G (2016). Statistical optimization of exopolysaccharide production by *Lactobacillus plantarum* NTMI05 and NTMI20. Int J Biol Macromol.

[7] Kajala I, Shi Q, Nyyssölä A, Maina NH, Hou Y, Katina K, Tenkanen M, Juvonen R (2015). Cloning and characterization of a *Weissella confusa* dextransucrase and its application in high fibre baking. PLoS ONE.

[8] Dilna SV, Surya H, Aswathy RG, Varsha KK, Sakthikumar DN, Pandey A, Nampoothiri KM (2015). Characterization of an exopolysaccharide with potential health-benefit properties from a probiotic Lactobacillus plantarum RJF4. LWT-Food Sci Technol.

[9] Zhang G, Zhang W, Sun L, Sadiq FA, Yang Y, Gao J, Sang Y (2019). Preparation screening, production optimization and characterization of exopolysaccharides produced by Lactobacillus sanfranciscensis Ls-1001 isolated from Chinese traditional sourdough. Int J Biol Macromol..

[10] Zhang Y, Dai X, Jin H, Man C, Jiang Y (2021). The effect of optimized carbon source on the synthesis and composition of exopolysaccharides produced by *Lactobacillus paracasei*. J Dairy Sci.

[11] Rajoka MSR, Mehwish HM, Kitazawa H, Barba FJ, Berthelot L, Umair M, Zhu Q, He Z, Zhao L (2022). Techno-functional properties and immunomodulatory potential of exopolysaccharide from *lactiplantibacillus plantarum* MM89 isolated from human breast milk. Food Chem.

[12] Wei L, Mao Y, Liu H, Ke C, Liu X, Li S (2022). Effect of an inorganic nitrogen source (NH4) 2SO4 on the production of welan gum from *Sphingomonas* sp. mutant obtained through UV-ARTP compound mutagenesis. Int J Biol Macromol.

[13] Bengoa AA, Llamas MG, Iraporda C, Dueñas MT, Abraham AG, Garrote GL (2018). Impact of growth temperature on exopolysaccharide production and probiotic properties of *Lactobacillus paracasei* strains isolated from kefir grains. Food Microbiol.

[14] Suryawanshi N, Naik S, Eswari JS (2019). Extraction and optimization of exopolysaccharide from *Lactobacillus sp* using response surface methodology and artificial neural networks. Prep Biochem Biotechnol.

[15] Kuo HC, Kwong HK, Chen HY, Hsu HY, Yu SH, Hsieh CW, Lin HW, Chu YL, Cheng KC (2021). Enhanced antioxidant activity of *Chenopodium Formosanum Koidz* by lactic acid bacteria: optimization of fermentation conditions. PLoS ONE.

[16] Bower JA. Statistical methods for food science: Introductory procedures for the food practitioner. 2nd Edition. Wiley; 2013. ISBN: 978-1-118-54162-3.

[17] Lee KM, Gilmore DF (2005). Formulation and process modeling of biopolymer (polyhydroxyalkanoates: PHAs) production from industrial wastes by novel crossed experimental design. Process Biochem.

[18] Elmansy EA, Elkady EM, Asker MS, Abdou AM, Abdallah NA, Amer SK (2022). Exopolysaccharide produced by *lactiplantibacillus plantarum* RO30 isolated from Romi cheese: characterization, antioxidant and burn healing activity. World J Microbiol Biotechnol.

[19] Dubois M, Gilles KA, Hamilton JK, Rebers PA, Smith F (1956). Colorimetric Method for Determination of Sugars and related substances. Anal Chem.

[20] Ahmed Z, Mehmood T, Ferheen I, Noori AW, Almansouri M, Waseem M (2023). Optimization of exopolysaccharide produced by *L. Kefiranofaciens* ZW3 using response surface methodology. Int J Food Prop.

[21] Wang Y, Li C, Liu P, Ahmed Z, Xiao P, Bai X (2010). Physical characterization of exopolysaccharide produced by *Lactobacillus plantarum* KF5 isolated from Tibet Kefir. Carbohydr Polym.

[22] Shene C, Bravo S (2007). Whey fermentation by *Lactobacillus delbrueckii* subsp. bulgaricus for exopolysaccharide production in continuous culture. Enzyme Microb Technol.

[23] Adesulu-Dahunsi AT, Jeyaram K, Sanni AI, Banwo K (2018). Production of exopolysaccharide by strains of *Lactobacillus plantarum* YO175 and OF101 isolated from traditional fermented cereal beverage. Peer J.

[24] Mozzi F, Rollan G, De Giori GS, De Valdez GF (2001). Effect of galactose and glucose on the exopolysaccharide production and the activities of biosynthetic enzymes in *Lactobacillus casei* CRL 87. J Appl Microbiol.

[25] Zannini E, Waters DM, Coffey A, Arendt EK (2016). Production, properties, and industrial food application of lactic acid bacteria-derived exopolysaccharides. Appl Microbiol Biotechnol.

[26] Adesulu-Dahunsi AT, Sanni AI, Jeyaram K (2018). Production, characterization and in vitro antioxidant activities of exopolysaccharide from *Weissella cibaria GA44*. LWT-Food Sci Technol.

[27] Gancel F, Novel G (1994). Exopolysaccharide production by *Streptococcus salivarius* ssp. *thermophilus* cultures. 1. Conditions of production. J Dairy Sci.

[28] Gamar LK, Blondeau, Simonet JM (1997). Physiological approach to extracellular polysaccharide production by *Lactobacillus rhamnosus strain* C83. J Appl Microbiol.

[29] Serrato RV, Sassaki GL, Gorin PA, Cruz LM, Pedrosa FO, Choudhury B, Carlson RW, Iacomini M (2008). Structural characterization of an acidic exoheteropolysaccharide produced by the nitrogen-fixing bacterium *Burkholderia tropica*. Carbohydr Polym.

[30] Zhang L, Zhao B, Liu CJ, Yang E (2020). Optimization of biosynthesis conditions for the production of exopolysaccharides by *Lactobacillus plantarum SP8* and the exopolysaccharides antioxidant activity test. Indian J Microbiol.

[31] Shi T, Aryantini NPD, Uchida K, Urashima T, Fukuda K (2014). Enhancement of exopolysaccharide production of Lactobacillus fermentum TDS030603 by modifying culture conditions. Bioscience of Microbiota food and Health.

[32] Bhat B, Vaid S, Habib B, Bajaj BK (2020). Design of experiments for enhanced production of bioactive exopolysaccharides from indigenous probiotic lactic acid bacteria. Indian J Biochem Biophys.

[33] Ismail B, Nampoothiri KM (2010). Production, purification and structural characterization of an exopolysaccharide produced by a probiotic *Lactobacillus plantarum* MTCC 9510. Arch Microbiol.

[34] Aslim B, Yuksekdag ZN, Beyatli Y, Mercan N (2005). Exopolysaccharide production by *Lactobacillus delbruckii* subsp bulgaricus and *Streptococcus thermophilus* strains under different growth conditions. World J Microb Biot.

[35] Degeest B, Vaningelgem F, De Vuyst L (2001). Microbial physiology, fermentation kinetics, and process engineering of heteropolysaccharide production by lactic acid bacteria. Int Dairy.

[36] Khanh TBTD, Thao DTT (2016). Optimal conditions for exopolysaccharide production by *Lactobacillus plantarum* T10. J Sci Technol.

[37] Haj-Mustafa M, Abdi R, Sheikh-Zeinoddin M, Soleimanian-Zad S (2015). Statistical study on fermentation conditions in the optimization of exopolysaccharide production by *Lactobacillus rhamnosus* 519 in skimmed milk base media. Biocatal Agric Biote.

[38] Wang X, Shao C, Liu L, Guo X, Xu Y, Lü X (2017). Optimization, partial characterization and antioxidant activity of an exopolysaccharide from *Lactobacillus plantarum* KX041. Int J Biol Macromol.

[39] Polak-Berecka M, Wasko A, Kubik-Komar A (2014). Optimization of Culture conditions for Exopolysaccharide production by a probiotic strain of *Lactobacillus rhamnosus* E/N. Pol. J Microbiol.

[40] Dertli E, Mayer MJ, Narbad A (2015). Impact of the exopolysaccharide layer on biofilms, adhesion and resistance to stress in *Lactobacillus johnsonii* FI9785. BMC Microbiol.

[41] Živković M, Miljković MS, Ruas-Madiedo P, Markelić MB, Veljović K, Tolinački M, Soković S, Korać A, Golić N (2016). EPS-SJ Exopolisaccharide produced by the strain Lactobacillus paracasei subsp. paracasei BGSJ2-8 is involved in adhesion to epithelial intestinal cells and decrease on E. Coli Association to Caco-2 cells. Front Microbiol.

[42] Oleksy-Sobczak M, Klewicka E, Piekarska-Radzik L (2020). Exopolysaccharides production by *Lactobacillus rhamnosus* strains–optimization of synthesis and extraction conditions. Lwt.

[43] Mıdık F, Tokatlı M, Bağder Elmacı S, Özçelik F (2020). Influence of different culture conditions on exopolysaccharide production by indigenous lactic acid bacteria isolated from pickles. Arch Microbiol.

[44] Sharma S, Sharma V, Nargotra P, Bajaj BK (2020). Bioprocess development for production of a process-apt xylanase with multifaceted application potential for a range of industrial processes. SN Appl Sci.

